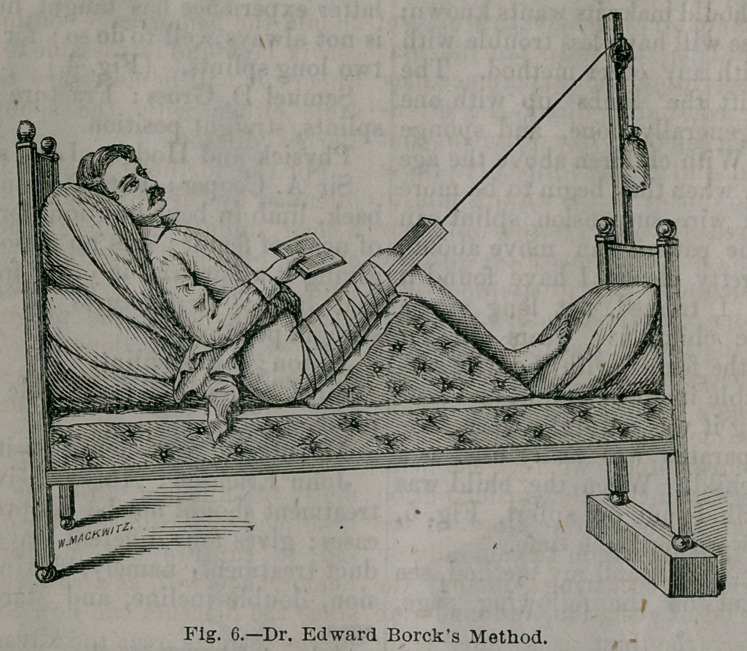# Review on the Treatment of Fracture of the Femur

**Published:** 1878-06-20

**Authors:** Edward Brock

**Affiliations:** Member of the Medical and Chirurgical Faceulty of Maryland and Baltimore Medical Associarion, Etc., Etc.


### REVIEW ON THE TREATMENT OF FRACTTRE OF THE FEMUR.

By Edward Brock, M.D., Member of the Medical and Chirurgical Faculty of Maryland and Baltimore Medical AssocNarion, Etc., Etc.

After long study and observation, I gave in the January number of the St. Louis *Medical and Surgical Journal*, my method of treating fracture of the femur in some cases; and advocated therein the double inclined plane, for reasons for which the reader is referred to the above number of the Journal. A marvelous coincidence brought the January number of the *Medical Record* of New York, into my hands, in which I find on the first page, a lecture on fracture of the shaft of the femur in children, by our distinguished surgeon, Frank H. Hamilton, M.D., with a wood cut illustrating his method, and advocating precisely the reverse of the course that I advocated. My method is intended only for youths and adults. For infants and small children it is not practicable. Dr. Hamilton's article refers to children only, but the fact that his opinion should be so directly reverse to my own, was to me of great interest. I, therefore, eagerly read and studied his article, to find, perhaps, my own mistake, for it is only by interchanging our views, and by giving each other the benefit of our experience, that we learn. It is this desire that induces me to write this paper, and bring before the profession a survey of the subject. In my article, mentioned above I pointed out why the long splints are not well adapted. The reason stated was, that the femur is not a straight bone from its head to the knee joint, and, therefore, the effort to keep it straight by pulling the leg outward, as is done with most of the long splints, more or less deformity must be produced. What is true as to the position of the femur in the adult, holds good in the child. Dr. Hamilton's splints, as Fig. 4 shows, is narrow above, and wider below, so as to pull the legs apart. He applies the same to children under thirteen years of age, and very correctly says: "Fractures in children are often transverse, denticulated, and especially in the very young only partially separated, not at all overlapping or greenstick. The muscles have no power to produce overlapping, and that in view of this fact the treatment should differ." Then he passes in review, different modes of applying splints, and is particularly disgusted with the double inclined plane, charging it with shortening and with other faults. In this cattegory he includes the lateral and coaptative splints, etc.

Dr. Hamilton then proceeds and says: "The straight position with short or coaptative splints, and the single long splint, with pulleys and weights, or such an apparatus as we have found best for adults, fail again in the case of infants and children." We will grant this.

He then describes his method, as shown in fig. 4. This method speaks for itself, and hardly needs explanation. Instead of one long splint there are two; they are widely separated below, which it is claimed will prevent, in some measure, the soiling of the cloths, by urine and fæces. There are short coaptative splints, pads, bandages, etc.; perineal band in most cases are used, and for six year-old children there are, in addition, pulleys and weights. Here we have a most complicated arrangement — the old-fashion long splint, with short coaptative splints combined. The gentleman takes great pains in describing all the details of this dressing; while it is true that upon details will depend the success of the result, particularly so ot an apparatus that is to be employed by others than the inventor, as the latter cannot be responsible for his invention, if it is not used correctly, yet these numerous details detract much from its usefulness.

But let us see whether it is actually necessary to encase a child or infant, in an apparatus like that which Dr. Hamilton recommends. I have never found such a confining method necessary, and feel sure this is the case with most surgeons, for these reasons: First, in greenstick fractures, which almost always occur on the inside of the femur, the outer half of the bone acts as a splint; a single coaptative splint and bandage is all that is needed; in such a case a little moving about by the patient can do no harm, while if the long splint be used, the legs drawn outward and kept straight we may do mischief; and this may be serious, which would be avoided if we allow a little more natural movement of the limbs. As we do not generally meet with oblique fracture in children, and generally have no contraction of muscle to overcome, we need no extension by pulleys and weights. If this is so, they are superfluous.

In denticulated fractures, it needs a little more care, but by no means does it need such squeezing and splinting as presented by Dr. Hamilton; at least this is my experience. With all due regard

Fig. 4, Illustrative of Dr. F. H. Hamilton's Method.

for the distinguished learning of the New York gentleman, for I do consider him as one of our highest surgeons, I, for one, would not be willing to try this splint in hardly any case, unless I learned from actual observation the good results that are claimed by the author.

The requirements of splints for any fracture, are that while they fulfill their purpose in maintaining the ends of the fractured bone in proper relation to each other, they should also keep the limb in as natural a position as possible. It should be light, easily put on and removed; all complicated apparatus render the treatment complicated, and as it is admitted by all surgical writers, that we, as a rule, have no shortening in very young children, it matters not whether we employ plaster, leather, starch or the suspension splint. This much is sure: tie a child up, harness it all over, and the more you put on, the harder the child will struggle to get it off, because of the uncomfortableness of the thing. Besides this, such is the nature of a child, that the less you bundle it up and the freer it has the use of its limbs, the sooner it will feel itself reconciled to the necessity of keeping comparatively quiet.

We seldom meet with a fractured femur in an infant. They happen mostly after the child begins to walk, but if we should meet them, they may be treated almost without any apparatus; simply tieing the legs together is about all there is required. With children under the age of five or six, I prefer the pasteboard splint, as follows: Take a piece of muslin or paper, fasten around the limb; for the purpose of cutting a pattern, put it

Fig. 5.—Pasteboard Splint.

upon a good piece of pasteboard, and you have it the shape as shown in Fig. 5; reverse it, take a ruler, and cut with a sharp knife $\frac{3}{4}$ of its thickness the lines indicated in Fig. 5. It is for left leg; "a—a" meets at the inside. It may also be formed to meet and open on the outside. Roll it up, and give it good coatings of shellac varnish, wrap up the limb in cotton batting, apply the splint, tie it with two or three ribbons or bandage, or punch holes and lace it; then tie the limbs together at the knees and the ankle, or bandage the limbs all the way up; put a soft pillow under the knees; then leave the child alone; it can be inspected every day with ease and comfort to the patient.

I had as good a result with this as with any other method. It is advisable to prepare two splints at once, so as to have one ready for change, as we well know that it is almost impossible to keep the bandages entirely clean, even if the child should make its wants known; yet the nurse will have less trouble with this, than with any other method. The nurse can lift the limbs up with one hand, as is generally done, and sponge the parts. With children above the age of six years, when they begin to be more rational, the wire suspension splint can be used; the patient can move about; also keep pretty clean; I have found it so at least. I treated, not long ago, a little idiotic child, five years old, for fracture of the femur, with the wire suspension double inclined splint, and notwithstanding it was naturally very restless, the apparatus was really used as a toy by the child. When the child was ready to walk I put on splint, Fig. 5, which was worn for some time.

To better understand my method, see the wood-cut on the following page, Fig. 6.

The risk of œdema is not greatly to be feared, if this method is employed, as the limbs rest upon a soft pillow, and the foot is not bound down, but can be moved and elevated at intervals. I will also observe, that special apparatuses may be well adapted to special cases, but that no one apparatus is applicable to every case.

There is nothing new under the sun. Petit, Heister, and Duverney recommended long ago the extending means to be applied just above the condyles of the os femoris. See Cooper's Dict., 1830. Let us examine the views and opinions of some of the different authors:

Albucasis: He used long splints, recmends the limb to be bandaged, and om the hollow places to be padded with soft material.

Paulus Aegineta: Patient to lie upon his back, the leg to be wrapped in a thick garment, and wool on each side to prevent moving the limb; a foot-board well curved to the foot, the whole covered with a skin.

Professor Frank H. Hamilton: In both his works on general surgery and treatise on fractures: Thigh bound to long side splints, but admits that the latter experience has taught him that it is not always well to do so; for children, two long splints. (Fig. 4.)

Samuel D. Gross: Fracture box with splints, straight position.

Physick and Hodge: Long splint.

Sir A. Cooper: Patient lying on his back, limb in bent position, for fracture of neck of femur; sees no reason for not giving it a fair trial in other fractures of that bone; in fractures of condyles, straight position.

Liston: Long splint.

Sir Charles Bell: Double inclined plane.

McIntyre: Semi-flexed position.

John Erichson: An exclusive plan of treatment should not be adopted for all cases; gives four different ways to conduct treatment, namely, flexing, extension, double incline, and starch bandages.

John Ashhurst, jr., has never seen a perfect cure; considers one-half to one inch a satisfactory result; thinks the weight and extension apparatus the most convenient.

Pott: Limb on its side, knee bent.

Billroth: Plaster of Paris splint; says the more practice one has applying them the more rarely will bad results happen.

Ferguson: Straight splint.

Gosselin: Surg. Dis. of youth. Points out that patients cannot lie squarely on their backs; the attempt to do so produces pain; that shortening always exists in adults; employs Scultet apparatus, semi-flexion; also, uses Honnequin's splint; uses extension, and prefers the movable bandages; says that none of the continuous extension apparatuses have taken rank in the practice.

Sanson: Semi-flexion.

Holmes: Children's fractures heal without any perceptible shortening or deformity; the treatment simply consists in rest on a splint, with knee and hip bent.

Guersant: Simple fractures in children heal without difficulty and deformity; if there is deformity, time modifies it, for men have presented themselves with a proven record that their femur had been broken when a child, and yet, when examined, it could not have been decided, in many cases, that any fracture had existed. Nearly all of those individuals were fit for military duty; employs Dupuytren's method.

G. R. Parkes: Metallic fracture splints; long extension and counter extension by tubes or rods; claims no shortening; the fracture can be examined without interfering with this apparatus. It is a neat contrivance, and appears to be preferable to any of the old style long splints.

Professor C. Heine, Insbruck: Plaster of Paris.

The reader is also referred to Dr. Cowling's paper on fractures, read before the Central Kentucky Medical Association, last July, which contains valuable points.

RESUME.

1. That the long splint has been used since time immemorial; the inclined plane also.

2. That no apparatus is perfect, and none answers for all cases, but all have their advantages and faults more or less, and each may serve well in special cases.

3. That we will have more or less shortening in adults, no matter what the treatment may have been; shortening rarely happens in children, for the great and wise doctor, Nature, comes in time to our assistance and corrects our shortcomings.

4. That it is not prudent to confine ourselves exclusively to one apparatus, but must admit that the surgeon who has had an extensive practice and experience with a particular apparatus will obtain better results with it than he who applies it only occasionally.—*St. Louis Med. and Surg. Journal.*

## Figures and Tables

**Fig. 4. f1:**
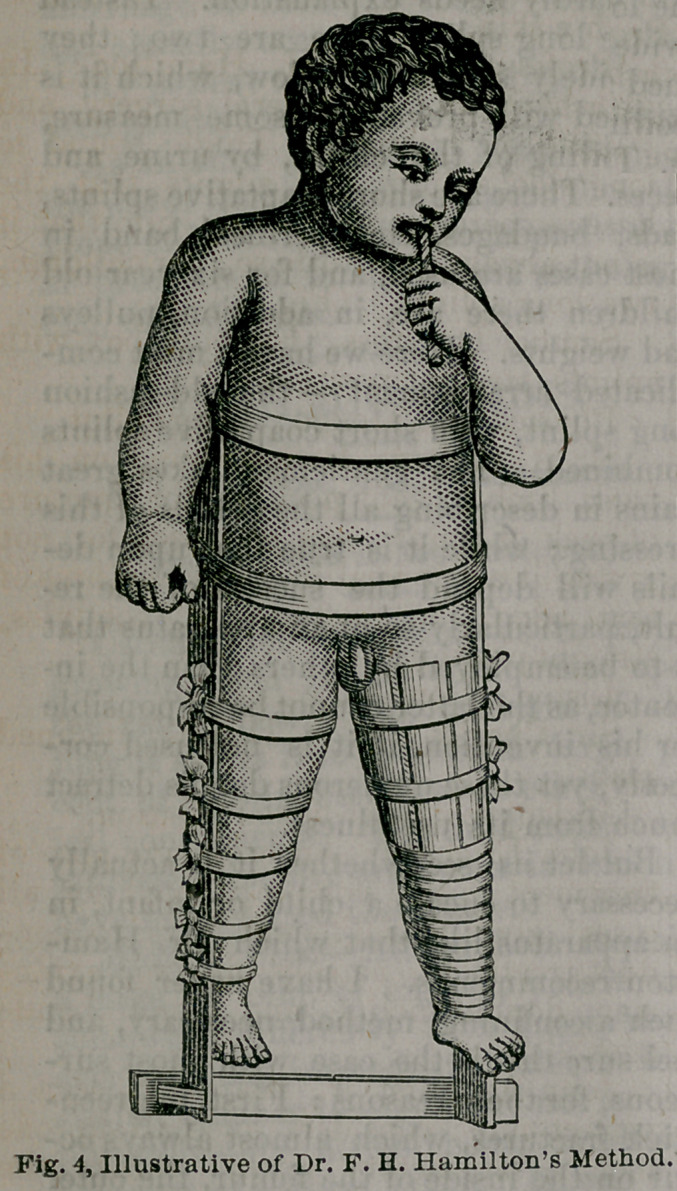


**Fig. 5. f2:**
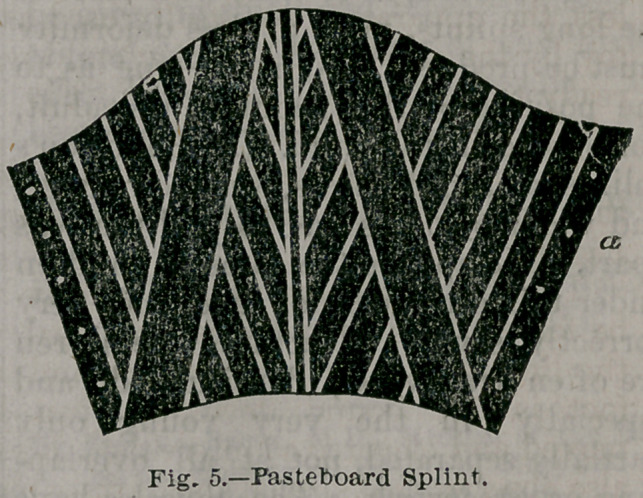


**Fig. 6. f3:**